# Plasma Fibronectin as a Novel Predictor of Coronary Heart Disease: A Retrospective Study

**DOI:** 10.3390/jcdd10100415

**Published:** 2023-10-02

**Authors:** Longyun Peng, Haiwei Deng, Jie Li, Guihua Lu, Yuan-Sheng Zhai

**Affiliations:** 1 Department of Cardiology, The First Affiliated Hospital, Sun Yat-Sen University, Guangzhou 510800, China; penglyun@mail.sysu.edu.cn (L.P.); denghw26@mail.sysu.edu.cn (H.D.); lijie268@mail.sysu.edu.cn (J.L.); 2 Key Laboratory on Assisted Circulation, Ministry of Health, Guangzhou 510800, China

**Keywords:** atherosclerosis, acute myocardial infarction, fibronectin, coronary heart disease, biomarker

## Abstract

Although fibronectin has been associated with the pathogenesis of atherosclerosis, little is currently known about the relationship between plasma fibronectin and coronary heart disease (CHD). This retrospective study aimed to determine the predictive value of plasma fibronectin for CHD and its severity. A total of 1644 consecutive patients who underwent selective coronary angiography were recruited into the present study. The characteristics and results of the clinical examination of all patients were collected. Logistic regression analyses were performed to determine the predictive value of plasma fibronectin for the presence and severity of CHD. Compared with non-CHD patients, the CHD patients showed significantly higher plasma levels of troponin I and creatine kinase isoenzyme, along with lower plasma levels of fibronectin. However, no significant differences were detected in plasma fibronectin among patients with different grades of CHD. The logistic regression model showed that plasma fibronectin remained an independent predictor of CHD after adjustment with a 1.39-fold increased risk for every 1 SD decrease in plasma fibronectin. Nevertheless, plasma fibronectin could not predict the severity of CHD determined by the number of stenosed vessels and the modified Gensini score. This study demonstrated that lower plasma fibronectin might be an independent predictor of CHD, but it may be of no value in predicting the severity of CHD.

## 1. Introduction

Coronary heart disease (CHD) is one of the leading causes of death worldwide, and it represents a major global public health burden [1]. Almost 11,390,000 Chinese individuals are affected by CHD, with a high CHD-related mortality rates since 2012 [2]. Thus, identifying CHD patients who may benefit from early intervention is essential to improve patient prognosis. Atherosclerosis is reportedly the most common etiological factor of CHD. It is widely acknowledged that various cellular processes contribute to the development of atherosclerosis. Current evidence suggests that fibronectin (FN) may have a potential role in the pathogenesis of atherosclerosis [3].

It is well established that FN is encoded via a gene of over 75 kb in length, located on chromosome 2q34. As a polymorphic and high-molecular-weight glycoprotein, FN exists in a soluble form in plasma and other body fluids, as well as in an insoluble form in most tissues [4,5]. Its variants originate from post-translational modifications; alternative RNA splicing of the V, EIIIA, and EIIIB segments; and variable conformations, depending on environmental conditions [4,6,7]. An increasing body of evidence suggests that FN promotes cell–cell and cell–matrix interactions, thus playing an important role in diverse processes, including cell growth, adhesion, migration, and wound repair [8].

Plasma fibronectin (pFN), the soluble form of FN, is present at rather high concentrations in plasma and lacks the EIIIA and EIIIB segments found in tissue FN, called cellular fibronectin (cFN) [4]. Vascular endothelial cells and hepatocytes are reportedly the major sources of pFN, although it is also synthesized by various cells in vitro, including fibroblasts, smooth muscle cells, and epithelial cells [9]. FN is reportedly prominent in atherosclerotic lesions of the intima and promotes the transformation of the vascular smooth muscle cell (VSMC) from the contractile phenotype to the synthesizing phenotype in atherosclerotic lesions, indicating the role of FN in the development of atherosclerosis [10,11]. Consequently, it was suggested that pFN might be a predictor of CHD. Fatih et al. reported that pFN levels were higher in patients with CHD, but did not show any correlation with the severity of CHD [12]. A study reported contrasting results, with no association between FN gene polymorphisms and CHD, and found significantly lower levels of pFN in patients with CHD compared with controls [13]. However, a single-center, case–control study of 187 non-consecutive patients showed that pFN levels were neither associated with CHD nor its severity [14].

Taken together, the relationship between pFN and CHD remains controversial. This present study sought to determine the predictive value of pFN for the presence and severity of CHD.

## 2. Patients and Methods

### 2.1. Study Population

We reviewed patient data between May 2015 and September 2020 from the First Affiliated Hospital of Sun Yat-Sen University. The enrollment criteria included the following: (1) underwent selective coronary angiography; (2) age ≥ 18 years; and (3) presented complete medical records (including at least baseline characteristics, pFN, and surgical procedures).

Patients with diseases affecting pFN levels were considered ineligible for this study. Hence, the exclusion criteria were patients who had undergone surgical procedures in the previous three months, patients with malignant tumors and connective tissue disease, patients with severe liver and kidney dysfunction, patients with thromboembolism and disseminated intravascular coagulation, and patients with active infections.

The study protocol was in accordance with the 1975 Declaration of Helsinki and was approved by the ethics committee of the First Affiliated Hospital of Sun Yat-Sen University (Guangzhou, China, 2022049). Written informed consent was not necessary because of the retrospective design of the study.

**Figure 1:**
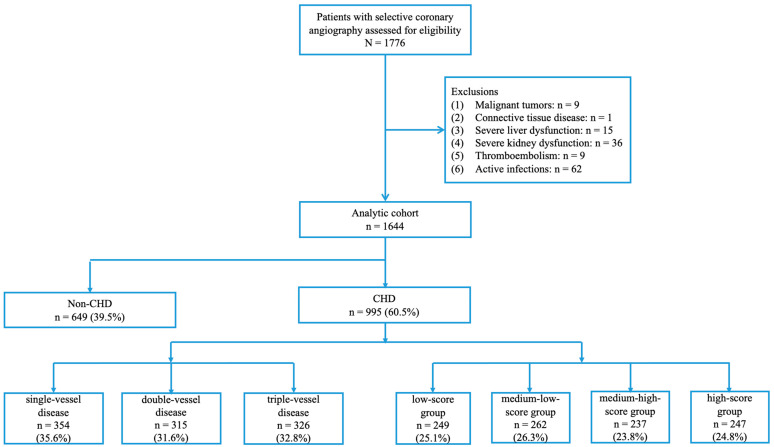
Study flow chart. CHD, coronary heart disease; low-score group, ≤25th percentile; medium-low-score group, >25th and ≤50th percentile; medium-high-score group, >50th and ≤75th percentile; high-score group, >75th percentile.

After exclusion, 1644 consecutive patients who underwent selective coronary angiography were recruited for the present study. Based on angiographic findings, 995 patients were assigned to the CHD group and 649 patients to the control group. To assess the grade of CHD, we first subdivided the CHD patients into single-vessel (n = 354), double-vessel (n = 315), and triple-vessel (n = 326) disease groups. We also categorized the CHD patients into low-score (≤25th percentile, n = 249), medium-low-score (>25th and ≤50th percentile, n = 262), medium-high-score (>50th and ≤75th percentile, n = 237), high-score (>75th percentile, n = 247) groups, based on the quartiles of the modified Gensini scores [15] (Figure 1).

### 2.2. Study Definition

CHD was defined by >50% stenosis in the internal diameter of the left main artery, left anterior descending artery, right or circumflex coronary artery, or their primary branches. In addition, patients who underwent coronary artery bypass graft surgery (CABG) or percutaneous coronary intervention (PCI), but who had no restenosis of the coronary artery, were also classified as CHD patients.

The severity of CHD was quantified using the modified Gensini score, as previously described [15], and the number of stenosed vessels, including single-vessel, double-vessel, and triple-vessel disease.

In this study, acute myocardial infarction (AMI) referred specifically to type 1, 4, and 5 myocardial infarction (MI). The diagnosis of type 1 MI required a rise and/or fall of troponin, with at least one value above the 99th percentile upper reference limit (URL) and with at least one of the following: symptoms of acute myocardial ischemia; new ischemic ECG changes; the development of pathological Q waves; imaging evidence of new loss of viable myocardium or new regional wall motion abnormality in a pattern consistent with an ischemic etiology; and the identification of a coronary thrombus via angiography, including intracoronary imaging. The diagnosis of type 4 and 5 MI, which were related to PCI or CABG, respectively, was also in accordance with the recommendation of the European Society of Cardiology (ESC) fourth universal definition of myocardial infarction [16]. Non-AMI cases consisted of angina, ischemic cardiomyopathy, or silent ischemia.

Acute heart failure (AHF) diagnosis was based on a history of cardiovascular disorders; clinical manifestations, such as hypertension, deteriorating dyspnea, severe edema, and rales; electrocardiography; N-terminal pro–B-type natriuretic peptide (NT-proBNP); and the results of chest X-ray and echocardiography, in accordance with the guidelines of the American Heart Association and European Society of Cardiology [17,18].

Blood pressure was measured three times, at rest and seated for 5 min. The average of these measurements was used for analysis. According to the 2020 International Society of Hypertension Global Hypertension Practice Guidelines and the 2018 Chinese Guidelines for Prevention and Treatment of Hypertension, hypertension was defined as systolic blood pressure ≥ 140 mmHg or diastolic blood pressure ≥ 90 mmHg, or current treatment with antihypertensive medication [19,20].

Diabetes mellitus was defined as a fasting plasma glucose (FPG) ≥ 126 mg/dL (7.0 mmol/L) or a 2 h plasma glucose (2 h PG) ≥200 mg/dL (11.1 mmol/L) during a 75 g oral glucose tolerance test (OGTT), based on the 2020 Standards of Medical Care in Diabetes recommended by American Diabetes Association (ADA) [21].

Hyperuricemia was defined as a fasting serum uric acid level (SUA) ≥7 mg/dL (420 μmol/L), according to the Guidelines for the Diagnosis and Management of Hyperuricemia and Gout in China (2019) [22].

### 2.3. Data Collection

A uniform data collection form was used to collect the following information for every patient: (1) demographic characteristics (such as age, gender, and body mass index (BMI)); (2) medical history (such as hypertension, diabetes mellitus, heart failure, and hypercholesteremia); (3) the results of biochemical indexes, including pFN, NT-proBNP, troponin I (CTnI), creatine kinase isoenzyme (CK-MB), triglyceride (TG), total cholesterol (TC), low-density lipoprotein cholesterol (LDL-C), and white blood cells (WBC); and (4) angiographic information (such as multivessel artery lesions, the location of the target lesion, and the stenosis degree of target lesion).

### 2.4. Biomarker Assays

Overnight fasting venous blood samples were obtained from all subjects the day after admission. The levels of pFN, NT-proBNP, CTnI, CK-MB, TG, TC, LDL-C, and WBC were determined following standard laboratory procedures. The levels of pFN, NT-proBNP, and CTnI were measured using a chemiluminescent immunoassay (Automated Immunoassay Analyzer, Siemens Healthcare Diagnostics Inc., Munich, Germany). Plasma levels of other biomarkers were analyzed using conventional methods.

### 2.5. Coronary Angiography

Coronary angiography was performed using standard Judkins techniques. All coronary angiograms were reviewed by two experienced invasive cardiologists blinded to patient clinical characteristics. The stenosis severity was determined via visual and digital quantification of the luminal diameter. Points of disagreement on the severity of stenosis were resolved via discussion with a third cardiologist.

### 2.6. Statistical Analysis

The normality of continuous variables was analyzed using the Kolmogorov–Smirnov test. Variables that were normally distributed were expressed as mean ± standard deviation, or as median and interquartile range, if they were not normally distributed. Proportions were used to express categorical variables. Depending on the normality of the distribution, a two-sample Student’s *t*-test or a non-parametric test was used to compare continuous variables between two groups. Categorical variables were compared using Chi-squared tests. Multiple comparisons between study groups were performed using the Kruskal–Wallis test, followed by Bonferroni analysis or one-way analysis of variance (ANOVA) and a post hoc test. Binary logistic regression was performed to evaluate the predictive value of pFN for CHD and the severity of CHD, and to calculate the odds ratio (OR) and 95% confidence interval (CI). A *p*-value < 0.05 for two-tailed tests was considered statistically significant. The Statistical Package for Social Science (SPSS) for Windows, version 22.0 (IBM, Almond, New York, NY, USA), and Stata 16 were used for statistical analyses.

## 3. Results

### 3.1. Clinical Characteristics of the Study Population

The clinical characteristics of the participants are shown in Table 1. A total of 1644 consecutive inpatients were recruited in this present study, including 995 CHD patients and 649 non-CHD patients. The CHD patients were more likely to be older than the non-CHD patients. In addition, CHD patients consisted of a significantly higher proportion of males (67.3% vs. 50.4%). Compared with non-CHD patients, patients with CHD were more likely to suffer from hypertension (70.1% vs. 49.5%), diabetes mellitus (40.4% vs. 17.3%), and AHF (39.0% vs. 6.62%). No significant difference was found in the proportion of patients with hyperuricemia between the two groups.

In comparison with patients with single-vessel disease, patients with double-vessel or triple-vessel disease exhibited higher rates of hypertension, diabetes mellitus, AMI, and AHF. Additionally, the incidence of AMI increased with the number of stenosed vessels. However, we did not observe significant differences in sex, BMI, hyperuricemia, and history of MI or PCI among single-vessel, double-vessel, and triple-vessel disease groups.

As one of the most widely used angiographic scoring systems, the Gensini score was calculated to estimate the grade of CHD. Patients in the high-score group were more likely to be male and older than patients in the other groups. Compared with the low-score patients, the high-score patients were more likely to suffer have diabetes mellitus, AMI, AHF, and a history of MI or PCI.

### 3.2. Plasma Biomarkers

The plasma levels of blood biomarkers are presented in Table 2. The plasma levels of FN in the CHD group were significantly lower than in the non-CHD group (190.2 (174.1, 205.7) mg/L vs. 197.2 (185.1, 211.5) mg/L). Compared with non-CHD patients, CHD patients had significantly higher plasma levels of CTnI and CK-MB.

In the subgroup analysis of CHD patients, the pFN levels did not differ between CHD patients with various numbers of stenosed vessels or varying Gensini scores. The plasma levels of CTnI and CK-MB associated with triple-vessel disease were significantly higher than those associated with single-vessel and double-vessel disease. Similarly, the plasma levels of CTnI and CK-MB were elevated with the increase in the Gensini score. However, all subgroups exhibited comparable plasma levels of TG, TC, and LDL-C.

### 3.3. Predictive Value of pFN for CHD

**Figure 2:**
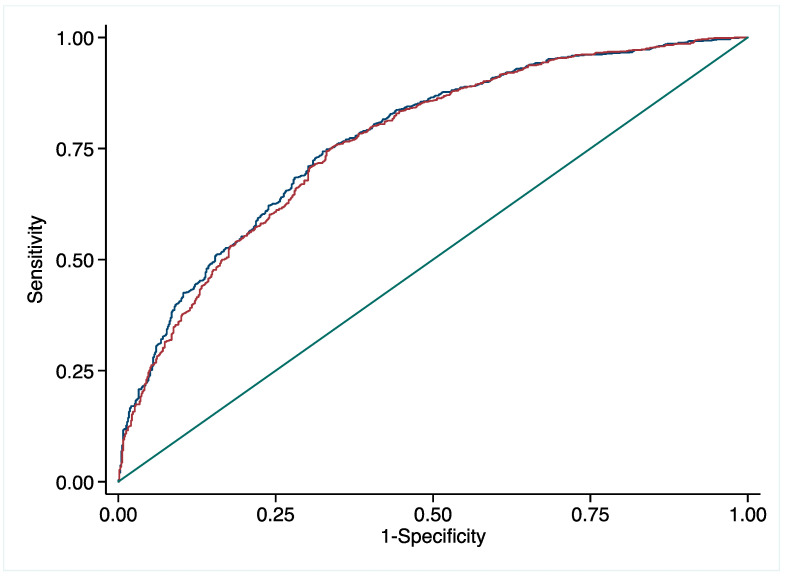
ROC curve analyses for predictive values of plasma fibronectin. Blue line represented the area under the curve (AUC) with plasma fibronectin and Red line represented the AUC without plasma fibronectin; The AUC, with and without plasma fibronectin, was 0.772 (95% CI 0.749–0.795) and 0.764 (95% CI 0.740–0.787), respectively.

**Figure 3:**
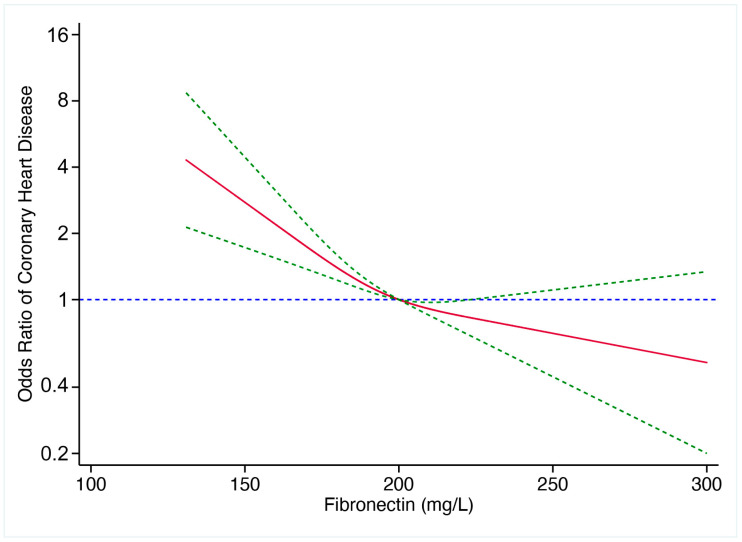
Relationship between plasma fibronectin and the odds ratio of coronary heart disease. Red line represented estimates of odds ratios and blue line represented 95%CI. The risk of coronary heart disease gradually increased with the decrease in plasma fibronectin.

Binary logistic regression was employed to determine the predictive value of pFN for CHD. Logistic regression showed that pFN, age, sex, TG, diabetes, hypertension, and hyperuricemia were independent predictive factors for CHD (Table 3). When confounding factors for CHD, such as age, sex, TG, TC, LDL-C, diabetes, hypertension, and hyperuricemia, were included in the logistic regression model, pFN remained an independent predictor for CHD. Since several factors were identified as independent predictive factors for CHD via multivariate logistic regression, we conducted an ROC curve analysis to evaluate the predictive values of pFN. ROC curves showed that the area under the curve (AUC), with and without pFN, was 0.772 (95% CI 0.749–0.795) and 0.764 (95% CI 0.740–0.787), respectively, indicating that pFN could independently predict CHD risk (Figure 2). As shown in Figure 3, further analysis revealed that the risk of CHD gradually increased with the decrease in pFN. A 1.39-fold increased risk for CHD was observed for every 1 SD decrease in plasma fibronectin (95% CI 1.22–1.59). Lastly, we found that an optimized pFN cut-off value to predict CHD was 183 mg/L.

### 3.4. Predictive Value of pFN for the Severity of CHD

We performed logistic regression to assess the predictive value of pFN for the severity of CHD, which was determined using the number of stenosed vessels and the Gensini score. In our study, the CHD patients were subdivided into three groups (patients with single-vessel, double-vessel, and triple-vessel disease, respectively), based on the involved coronary artery, or four groups (low-score, medium-low-score, medium-high-score, high-score group, respectively), based on quartiles of modified Gensini score. Logistic regression analysis demonstrated that pFN was not associated with the number of stenosed vessels or the level of the Gensini score when confounding factors such as age, sex, TG, TC, LDL-C, diabetes mellitus, hypertension, and hyperuricemia were included in the analysis, indicating that pFN may not independently predict the severity of CHD.

## 4. Discussion

In the present retrospective study, we explored pFN’s predictive value for atherosclerotic CHD and its severity. We found that pFN levels were lower in CHD patients than in non-CHD patients. Logistic regression and ROC curve analysis showed that pFN might be an independent predictor of CHD risk. In addition, a 1.39-fold increased risk for CHD was observed for every 1 SD decrease of plasma fibronectin and an optimized pFN cut-off value of 183 mg/L could predict CHD, indicating that pFN may be used as a screening marker for CHD in clinical practice when it is below 183 mg/L. However, a lower pFN level could not predict the number of stenosed coronary arteries and Gensini score, implicating that pFN might not be an independent predictor for the severity of CHD.

CHD remains an enormous worldwide socio-economic burden associated with extremely high morbidity and mortality. Atherosclerosis is well-established as the most important pathophysiological basis of CHD and is believed to be an inflammatory disease of large arteries characterized by the accumulation of lipids and extracellular matrix (ECM) proteins in the affected vessel wall [23]. The lesions of atherosclerosis represent a series of highly specific cellular and molecular responses: (1) the transition of isolated macrophages into foam cells and the deposition of lipids in the arterial intima; (2) VSMC migration from the media to the intima and inappropriate proliferation within the intima; and (3) the aggregation of platelet and/or fibrin in the intima [24]. A change in phenotype expression from “contractile” to “synthetic” initiates VSMC proliferation, which is characterized by a change in protein expression and the formation of extensive rough endoplasmic reticulum and a large Golgi complex, followed by migration from the media to the intima, finally leading to the accumulation of new ECM [25]. The synthetic phenotype of VSMC, proliferation, and migration are key elements in the formation of atherosclerotic plaques. The changes in VSMC in the process of atherosclerosis are regulated by a variety of growth factors and cytokines, such as fibroblast growth factor (FGF), interleukin-1 (IL-1), and FN [26].

FN was first identified as a “cold-insoluble globulin” during the purification of fibrinogen from human plasma in 1948 and named “fibronectin” in 1976 by Vaheri to imply its association with fibrinogen and fibrin [27,28]. FN is a dimeric glycoprotein consisting of two 250-kDa subunits linked by two C-terminal disulfide bonds. All FN molecules are composed of the same basic functional domains. Each subunit consists of a series of repeated modules: 12 type I modules, 2 type II modules, 15 to 17 type III modules, and a variable (V) sequence that is not homologous to other parts of FN. The repeated modules are arranged into several domains that bind fibrin, heparin, collagen, glycosaminoglycans, and cellular receptors. Type III can also participate in alternative splicing, including ED-A and ED-B (ED for “extradomain”). It has been established that the alternative splicing of ED-A, ED-B, and the variable region produces approximately 20 monomeric isoforms in humans, which can be categorized into pFN and cFN [28]. FN is now considered an indicator of ECM deposition in atherosclerotic processes and may participate in the pathogenesis of CHD, as characterized by the formation of coronary atherosclerotic plaque [29]. It reportedly serves as a bridge between cells and the interstitial collagen meshwork, greatly influencing the migration and proliferation of VSMC during atherosclerosis progression [30,31]. In addition, FN might contribute to the transformation of VSMC from the contractile phenotype to a synthetic phenotype. The synthetic phenotype, migration, and proliferation of VSMC are essential factors affecting coronary atherosclerosis and restenosis [25]. Consequently, pFN, the soluble form of FN, has huge prospects for application as a marker of CHD.

However, a great deal of controversy surrounds the association between pFN and CHD, with conflicting results reported regarding pFN levels in CHD patients. Our study showed that the pFN concentration is significantly decreased in CHD patients compared with the concentrations in non-CHD patients, implying that pFN may be an independent predictor of CHD. However, significantly lower or higher levels of circulating FN in CHD have been reported in the literature [12,13], which may be attributed to the following. First, differences in exclusion criteria may lead to inconsistencies in the results. In the present study, patients with AMI or AHF were included, while most previous studies excluded them. Moreover, the timing of pFN measurements might significantly influence the levels of pFN. Moreover, differences in ethnic populations may contribute to inconsistent findings, since studies have been conducted in various countries, including Turkey, Korea, the USA, and China. Finally, to our knowledge, the present study included the largest number of enrolled patients so far, which increased the robustness of our findings.

The exact mechanism of decreased pFN in CHD remains unclear. It is widely believed that decreased pFN levels may be attributed to excessive pFN consumption in the process of atherosclerosis. Indeed, pFN is leaked from the damaged endothelium and subsequently deposited on the affected vascular wall, promoting atherosclerotic lesion development [32]. In addition, participation in platelet adhesion and aggregation during blood clotting can result in the consumption of pFN in plaque rupture or erosion, characterized by AMI [10]. It is also possible that another mechanism might be associated with the involvement of pFN in reticuloendothelial or macrophage clearance of bacterial and non-bacterial particles under inflammatory conditions such as CHD [33].

Moreover, we revealed that lower pFN levels could not independently predict the severity of CHD quantified by the number of stenosed vessels and the level of the Gensini score, which was inconsistent with the results of some works in the literatures. One study reported that pFN levels in triple-vessel disease were significantly higher than in the control group, and that there was a strong correlation between pFN and severity of disease determined by the number of stenosed coronary arteries [34]. However, the study population was relatively small and might be subject to type I statistical errors.

There are several limitations in our study. Given the retrospective nature of our research, a prospective study is warranted to validate our results. In addition, selective bias might affect the results of our study, to a certain extent, since this was a single-center study. Finally, the current study revealed that pFN was not an independent predictor for the severity of disease, as determined by the number of stenosed coronary arteries. In the future, however, other indicators of CHD severity may be used, such as AMI and AHF.

## 5. Conclusions

In conclusion, our study demonstrated that lower pFN levels might be an independent predictor of CHD risk. However, pFN levels do not reflect the severity of CHD. Accordingly, incorporating pFN levels into the diagnostic algorithm of CHD may be recommended in the future.

## Figures and Tables

**Table 1:** Patient clinical characteristics.

	Non-CHD	CHD	Single	Double	Triple	Low	Medium-Low	Medium-High	High
	n = 649	n = 995	n = 354	n = 315	n = 326	n = 249	n = 262	n = 237	n = 247
Age (years)	59 (50, 67.5)	67 (58, 74) ^a^	66 (56, 74)	68 (57, 75)	67 (59, 74)	68 (60, 75)	68 (60, 76)	66 (57, 74) ^d,e^	66 (56, 73) ^d,e^
SEX, (%)									
Male	327 (50.4)	670 (67.3) ^a^	231 (65.3)	216 (68.6)	223 (68.4)	143 (57.4)	183 (69.8) ^d^	163 (68.8) ^d^	181 (73.3) ^d,e,f^
Female	322 (49.6)	325 (32.7)	123 (34.7)	99 (31.4)	103 (31.6)	106 (42.6)	79 (30.2) ^d^	74 (31.2) ^d^	66 (26.7) ^d,e,f^
BMI, (kg/m^2^)	23.6 (22.6, 24.6)	23.8 (22.5, 25.0)	23.5 (22.3, 24.9)	23.9 (22.7, 25.0)	23.8 (22.6, 24.8)	23.7 (22.6, 25.0)	23.9 (22.5, 24.9)	23.8 (22.4, 24.9)	23.7 (22.4, 24.9)
Hypertension (%)	321 (49.5)	697 (70.1) ^a^	222 (62.7)	224 (71.1)	251 (77.0) ^b^	164 (65.9)	195 (74.4)	158 (66.7)	180 (72.9)
Diabetes mellitus (%)	112 (17.3)	402 (40.4) ^a^	112 (31.6)	128 (40.6) ^b^	162 (49.7) ^b^	76 (30.5)	115 (43.9) ^d^	92 (38.8) ^d^	119 (48.2) ^d,e,f^
Hyperuricemia (%)	93 (14.3)	149 (15.0)	52 (14.7)	58 (18.4)	39 (12.0)	40 (16.1)	46 (17.6)	29 (12.2)	34 (13.8)
AMI (%)	0	282 (28.3) ^a^	61 (17.2)	84 (26.7) ^b^	137 (42.0) ^b,c^	13 (5.2)	56 (21.4) ^d^	87 (36.7) ^d,e^	126 (51.0) ^d,e,f^
AHF (%)	43 (6.62)	385 (39.0) ^a^	107 (30.2)	142 (45.1) ^b^	136 (41.7) ^b^	72 (28.9)	114 (43.5) ^d^	91 (38.4) ^d^	108 (43.7) ^d^
History of MI (%)	0	93 (9.35) ^a^	28 (7.91)	36 (11.4)	29 (8.90)	9 (3.6)	32 (12.2) ^d^	25 (10.5) ^d^	27 (10.9) ^d^
History of PCI (%)	0	203 (20.4) ^a^	61 (17.2)	75 (23.8)	67 (20.6)	30 (12.0)	71 (27.1) ^d^	56 (23.6) ^d,e^	46 (18.6) ^d,e^

CHD, coronary heart disease; single, single-vessel disease group; double, double-vessel disease group; triple, triple-vessel disease group; low, low-score group (≤25th percentile); medium-low, medium-low-score group (>25th and ≤50th percentile); medium-high, medium-high-score group (> 50th and ≤ 75th percentile); high, high-score group (>75th percentile). BMI, body mass index; AMI, acute myocardial infarction; AHF, acute heart failure; MI, myocardial infarction; PCI, percutaneous coronary intervention. Data were expressed as n (%) or median (interquartile range), unless noted otherwise. ^a^ *p* < 0.05 vs. non-CHD group; ^b^ *p* < 0.05 vs. single-vessel disease group; ^c^ *p* < 0.05 vs. double-vessel disease group; ^d^ *p* < 0.05 vs. low-score group; ^e^ *p* < 0.05 vs. medium-low-score group; ^f^ *p* < 0.05 vs. medium-high-score group.

**Table 2:** Plasma biomarkers.

	Non-CHD	CHD	Single	Double	Triple	Low	Medium-Low	Medium-High	High
	n = 649	n = 995	n = 354	n = 315	n = 326	n = 249	n = 262	n = 237	n = 247
pFN (mg/L)	197.2 (185.1, 211.5)	190.2 (174.1, 205.7) ^a^	189.8 (172.6, 206.0)	190.6 (175.9, 207.6)	190.1 (174.2, 204.6)	191.0 (174.6, 206.9)	188.6 (173.1, 205.9)	191.2 (176.6, 207.3)	190.2 (173.6, 203.0)
LDL-C (mmol/L)	3.06 (2.38, 3.73)	2.76 (2.09, 3.61) ^a^	2.73 (2.05, 3.52)	2.66 (1.96, 3.59)	2.87 (2.20, 3.68)	2.73 (2.07, 3.64)	2.64 (2.03, 3.35)	2.82 (2.11, 3.79)	2.86 (2.08, 3.69)
TC (mmol/L)	4.9 (4.1, 5.6)	4.5 (3.7, 5.4) ^a^	4.4 (3.7, 5.3)	4.5 (3.7, 5.6)	4.5 (3.7, 5.6)	4.5 (3.7, 5.5)	4.4 (3.6, 5.2)	4.5 (3.8, 5.7)	4.6 (3.7, 5.6)
TG (mmol/L)	1.38 (1.05, 1.98)	1.4 (1.0, 2.0)	1.33 (0.98, 1.85)	1.41 (0.99, 2.08)	1.45 (1.05, 2.09)	1.34 (1.00, 1.89)	1.26 (0.96, 1.96)	1.47 (1.06, 1.91)	1.50 (1.01, 2.23)
CTnI (pg/mL)	0.012 (0.01, 0.012)	0.012 (0.01, 0.42) ^a^	0.012 (0.01, 0.03)	0.012 (0.01, 0.2)	0.02 (0.012, 3.13) ^b,c^	0.012 (0.01, 0.02)	0.012 (0.01, 0.06) ^d^	0.013 (0.01, 6.69) ^d,e^	0.06 (0.01, 12.7) ^d,e,f^
CK-MB (ug/L)	0.6 (0.33, 1.2)	1.06 (0.55, 3.2) ^a^	0.84 (0.46, 2.04)	1.11 (0.54, 2.75) ^b^	1.44 (0.67, 5.96) ^b,c^	0.75 (0.43, 1.41)	0.92 (0.53, 2.23) ^d^	1.25 (0.61, 11.5) ^d,e^	1.8 (0.74, 18.1) ^d,e^
WBC (*10^9^/L)	6.41 (5.49, 6.94)	6.48 (5.68, 6.92)	6.48 (5.72, 6.86)	6.48 (5.64, 7.02)	6.48 (5.71, 7.18)	6.48 (5.75, 7.39)	6.48 (5.54, 6.83)	6.48 (5.60, 6.93)	6.48 (5.73, 7.19)

CHD, coronary heart disease; single, single-vessel disease group; double, double-vessel disease group; triple, triple-vessel disease group; low, low-score group (≤25th percentile); medium-low, medium-low-score group (>25th and ≤50th percentile); medium-high, medium-high-score group (>50th and ≤75th percentile); high, high-score group (>75th percentile). pFN, plasma fibronectin; LDL-C, low-density lipoprotein cholesterol; TC, total cholesterol; TG, triglyceride; CTnI, cardiac troponin I; CK-MB, creatine kinase isoenzyme; WBC, white blood cells. Data were expressed as median (interquartile range), unless noted otherwise. ^a^ *p* < 0.05 vs. non-CHD group; ^b^ *p* < 0.05 vs. single-vessel disease group; ^c^ *p* < 0.05 vs. double-vessel disease group; ^d^ *p* < 0.05 vs. low-score group; ^e^ *p* < 0.05 vs. medium-low-score group; ^f^ *p* < 0.05 vs. medium-high-score group.

**Table 3:** Analysis of factors predicting CHD risk.

Variables	OR	Lower 95% CI	Upper 95% CI	*p* Value
pFN	0.987	0.982	0.992	<0.001
Age	1.061	1.05	1.073	<0.001
TG Sex Hypertension	1.243 3.584 1.492	1.108 2.791 1.18	1.395 4.604 1.887	<0.001 <0.001 0.001
Diabetes mellitus	3.18	2.437	4.15	<0.001

CHD, coronary heart disease; pFN, plasma fibronectin; TG, triglyceride; OR, odds ratio; CI, confidence interval.

## Data Availability

Not applicable.
